# Association of *IL16* polymorphisms with periodontitis in Brazilians: A case- control study

**DOI:** 10.1371/journal.pone.0239101

**Published:** 2020-09-11

**Authors:** Victor Hugo Souza, Jeane Eliete Laguila Visentainer, Joana Maira Valentini Zacarias, Josiane Bazzo Alencar, Patrícia Yumeko Tsuneto, Cléverson Oliveira Silva, Samira Salmeron, Cristiane Maria Colli, Ana Maria Sell

**Affiliations:** 1 Post-Graduation Program in Biosciences and Physiopathology, Department of Clinical Analysis and Biomedicine, and Laboratory of Immunogenetics, State University of Maringá, Maringá, Paraná, Brazil; 2 Department of Basic Health Sciences, State University of Maringá, Maringá, Paraná, Brazil; 3 Department of Dentistry, State University of Maringá, Maringá, Brazil; 4 Ingá University Center, Maringá, Brazil; University of California San Francisco, UNITED STATES

## Abstract

Periodontitis (PD) is a chronic inflammatory process resulting from the relationship of the immune response with the components in dental plaque. Cytokines and their genetic polymorphisms seem to be involved in the immunopathogenesis of this disease. This study aimed to evaluate the correlation of *IL16* polymorphism with PD. A case-control study was conducted in a sample of individuals from southern Brazil. The genotyping of *IL16*, *rs11556218 T>G*, *rs4072111 C>T* e *rs4778889 T>C*, was performed using the PCR-RFLP methodology. The serum level of IL-16 was determined using an IL-16 ELISA kit for humans. SNPStats and OpenEpi software and Wilcoxon's U test were used to perform statistical analysis. *IL16 rs11556218* polymorphism was significantly associated to PD in nonsmoking patients: individuals with *G/G* genotype were less likely to develop PD compared to the *T/T* genotype (OR = 0.10; *Pc* = 0.019, codominant model). In addition, the *TTT* haplotype was associated with a high risk for PD (OR = 2.45; *P* = 0.01). A low IL-16 serum level was observed among individuals with PD when compared to controls (*P* = 0.027). Thus, the *IL16* rs16556218 polymorphism and the serum levels of IL-16 were associated with periodontitis in a Brazilian sample, and this was influenced by environmental factors such as smoking.

## Introduction

Periodontal disease is a complex and multifactorial disease characterized by the aggression of the periodontal ligament and alveolar bone [1]. This disease has a high prevalence that varies widely between countries and has an estimated average of 10.8% worldwide; it affects 743 million people and is considered the sixth most prevalent health condition in the world [2]. According to the International Workshop for a Classification of Periodontal Diseases and Conditions of 1999 [3], updated in 2017 [1, 4], periodontitis (PD) involves a chronic inflammatory process resulting from the individual's immune response against bacterial components present in tooth plaque [5]. This process may lead to functional and morphological compromises of the periodontium, and in more advanced cases, significant alveolar bone resorption and tooth loss [6]. Some diseases, genetic and environmental factors are involved in the development of this disease [7–9].

Cytokines play an important role in the pathogenesis of PD. Some of them, such as Interleukins (IL)-1β (IL-1β), Interleukin-6 (IL-6), and Tumor Necrosis Factor Alpha (TNF- ɑ) are activators of bone resorption through osteoclasts. These cytokines are also involved in neutrophil chemotaxis, the inflammatory process and the production of other pro-inflammatory cytokines [10–12]. They can be produced by monocytes after stimulation by other cytokines such as Interleucin-16 (IL-16) [13]. Little is known about the role of IL-16 in PD. However, IL-16 levels were lower in gingival crevicular fluid (GCF) of the teeth sites with PD compared to healthy tooth sites and this cytokine was correlated with the severity of disease in alcohol-consuming individuals and smokers [14].

IL-16, a chemokine initially described as a chemotactic factor for leukocytes, is produced by some cells, such as T lymphocytes, fibroblasts and dendritic cells [15]. This cytokine is associated with pleiotropic effects, exhibiting patterns of inflammatory or anti-inflammatory responses in diseases [16]. Its chemotactic role, especially for CD4+ cells, leads to cell recruitment and subsequently immune response. The gene for this cytokine, called *IL16*, is located on chromosome 15 (15q26.3) [17]. Polymorphisms in *IL16* have been associated with chronic inflammatory diseases and cancer in studies conducted in different populations, such as Chinese and Iranians [18–24].

Among the most studied polymorphisms are the single nucleotide polymorphisms (SNPs) consisting of the exchange of only one nucleotide in the gene sequence. These polymorphisms may interfere with the amount of the transcribed product (as described for SNP *rs4778889* of the *IL16*, present in the promoter region) or in the biological effect of the produced protein, when located in the exon region of the gene (such as *IL16 rs11556218* and *rs4072111*) [18]. *IL16* polymorphisms were previously studied in PD [23, 24]. Other SNPs in cytokine genes such as IL-1β, IL-2, IL-17 and IL-18 were associated with PD in our population from southern Brazil [25–27].

In an attempt to help elucidate the role of the genetic effect and cytokine production that affect the pathogenesis of PD, this study evaluated a possible association of *rs11556218 T>G*, *rs4072111 C>T* and *rs4778889 T>C* of *IL16* and the serum levels of IL-16 with PD in a Brazilian population. In addition, the relationship between these polymorphisms and PD in relation to gender, smoking habits and age of these individuals was evaluated.

## Materials and methods

### Sample selection

For this case-control study, 424 individuals were selected in the dental clinics of the State University of Maringá (DOD-UEM) and the Uningá University Center, between January 2012 and September 2018, both located in Maringá, northwest region of the state of Paraná, Brazil. This study was approved by the Ethics and Research Committee on Human Beings of the State University of Maringá (UEM-No. 719/2011, 2011 and 1,866,509, 2016). The subjects who met all the eligibility criteria for this study (case and controls) and agreed to participate were informed about its nature and signed the free and informed consent form.

All subjects were submitted to a periodontal clinical examination by a specialized periodontist who evaluated clinical parameters such as probing pocket depth (PPD), with an unc-15 periodontal probe (Hu-Friedy manufacturing, Chicago, IL, USA), clinical attachment level (CAL) and bleeding on probing (BOP) in four sites of each tooth (mesial, vestibular, distal and lingual). Clinical parameters were evaluated in order to classify individuals into case/control groups and the selection criteria was done according to the International Workshop for a Classification of Periodontal Diseases and Conditions of 1999 [3]. After the periodontal examination, participants were assigned to the case groups (n = 215) if they had at least 5 sites in non-adjacent teeth with PPD ≥ 5 mm, CAL ≥ 3 mm, and more than 25% BOP. The control group (n = 209) was formed by individuals without altered clinical insertion level, with a probing depth of ≤ 4 mm and with less than 25% bleeding on probing and plaque index of < 20%.

Although the criteria used for the classification of patients with periodontal disease was based on the 1999 classification [3], it contemplates the criteria for the definition of PD established in 2017 by the Workshop on the Classification of Periodontal and Peri-Implant Diseases and Conditions [1]. In this last classification, periodontitis was defined as the presence of at least 2 non-adjacent teeth with detectable interdental clinical attachment level (CAL), or detectable buccal or oral CAL ≥3 mm with pocketing ≥3 mm in ≥2 teeth, with observable CAL that cannot be attributed to causes not related to PD. In addition to that, in the recent classification, it is established that significant descriptions of PD should include the proportion of sites that bleed on the probing, the number of with PPD above certain limits (commonly ≥4 mm and ≥6 mm) and with CAL ≥3 mm and ≥5 mm: in our study we evaluated > 25% of BOP and at least 5 sites in non-adjacent teeth with PPD ≥ 5 mm and CAL ≥ 3 mm. Furthermore, for the classification of staging and grading [4], the patients in the present study may be included in stages II and III (based on severity, complexity and with extension and description of the distribution as localized or generalized) and grade B (moderate rate of progression).

Cases and controls were selected from adults over 30 years of age, with at least 20 teeth in the mouth, nonsmokers or smokers (including those who have smoked in the last 10 years). All individuals were considered ethnically mixed, due to the great miscegenation found in the Brazilian population. This classification was in accordance with the study previously conducted for the population of Paraná, southern Brazil, by Probst et al. [28] and confirmed for our region [29], whose population consisted mainly of Caucasians with European genetic origins (80.6%), with a lower presence of Africans (12.5%) and Amerindians (7.0%). Nevertheless, the self-declarations of ethnicity were collected according to the norms from the Brazilian Institute of Geography and Statistics (IBGE) (S1 Table), and no differences were found between the cases and controls indicating that there is no ethnic disproportion between them. To avoid possible confounding variables, pregnant women, Asians, individuals with chronic or acute inflammatory diseases, diabetes, cancer or autoimmune diseases, and those who had periodontal treatment in the last 6 months or who took antibiotics during this period were not included in our study.

### DNA extraction and sample collection

Peripheral blood samples were collected by venous blood puncture in a tube containing EDTA. DNA extraction from the samples was made from whole blood or buffy coat using the Salting Out method [30]. The DNA used for genotyping was diluted to a concentration of 50 ng/μl and stored at -20°C. The quality of the extracted DNA was analyzed by optical density in a Thermo Scientific Nanodrop 2000® (Wilmington, USA).

### Genotyping of *IL16* polymorphisms

The genotyping of *IL16* polymorphisms *(rs11556218*, *rs4778889 and rs4072111)* was performed according to Luo et al. [18] with modifications. Primer sequences were based on those described above [18] and tested in Primer Blast (NCBI) (https://blast.ncbi.nlm.nih.gov/Blast.cgi). Polymerase chain reaction (PCR) was performed in 10 μl with final concentration of 7.5 ng of DNA, 2.0 ng of each primer, 1.25 mM of MgCl2, 0.2 mM of dNTP (Invitrogen®, Frederick, MD, USA), 1X PCR buffer (5X Green Goat® Buffer, Promega, USA) and 0.5U of *Taq* Polymerase (GoTaq® Polymer DNA Polymerase, Promega, USA). The cycles were performed on the Veriti thermocycler (Applied Biosystems): five-minute cycle at 95°C; 35 cycles of 30 seconds at 95°C, 45 seconds at 61°C (for *rs11556218*), 67°C (for *rs4778889*) or 63°C (for *rs4072111*) and one minute at 72°C; at the end, 10-minute at 72°C. PCR products were digested for 16 hours at 37°C with *NdeI* digestion enzymes (Invitrogen®, Frederick, MD, USA) for *rs11556218*, *BsmAI* (Invitrogen®, Frederick, MD, USA) for *rs4072111* and *AhdI* (Invitrogen®, Frederick, MD, USA) for *rs4778889*. The results were observed by electrophoresis in 3.5% agarose gel with SYBR™ Safe (Invitrogen Life Technologies, Grand Island, NY, USA) with a run of 70V for 15min and then 90V for 35min, 300mA and 150W. The result was visualized under ultraviolet light. To ensure the quality of the results, we compared the frequency of the less frequent allele with other populations and analyzed whether the distribution of genotype frequencies for all analyzed SNPS was in accordance with Hardy-Weinberg equilibrium for the control group.

### Determination of the IL-16 serum levels

Serum levels of IL-16 were determined using an Enzyme-Linked Immunosorbent Assay (ELISA) kit (Sigma-Aldrich Co LLC, N°RAB0261, St. Louis, Missouri, USA) according to the manufacturer's instructions. The absorbance of each well was read in ELISA reader (ASYS HITECH GMBH—Eugendorf, Austria) using absorbance at 450 nm. The IL-16 serum levels (in pg/ml) of samples tested were estimated using the calculation of the standard curve. Reactions were made in duplicates.

### Statistical analysis

To obtain the minimum number of samples appropriate for this study with adequate statistical power (≥80%), the quantitative calculation software QUANTO [31] was used in the study design. At the significance level of 0.05, two-tailed test, K_P_ = 0.50, for Minor Allele Frequencies (MAFs) of *rs11556218*, *rs4072111* and *rs4778889*, power values of 93.2%, 80.5% and 92.2% were obtained, respectively, to detect a risk effect of 2.0, under a dominant model. Associations of *IL16* polymorphisms and descriptive analyses were performed using software SNPStats (https://www.snpstats.net/start.htm) [32] and the software OpenEpi version 3.01 (https://www.openepi.com/Menu/OE_Menu.htm). The association tests were based on linear or logistic regression according to the response variable. The analysis for SNPs was performed by choosing models of genetic inheritance (codominant, dominant, recessive, overdominant and log-additive) according to the Akaike information criterion (AIC) and adjusting for possible interactions with the studied covariates [32]. The *Odds Ratio* (OR) with confidence intervals (CI) of 95% was used. For the analysis of multiple SNPs, the Haploview and SNPStats software were used [32, 33] were used. The haplotype frequencies were determined using the expectation-maximization (EM) algorithm and the possible association of these haplotypes with PD was evaluated by the chi-squared test; furthermore, the linkage disequilibrium (LD) analysis between the SNPs and the permutation test for statistically significant results were performed. For statistically significant results, Bonferroni correction (BC) method was applied by multiplying the *P* values by the number of SNPs evaluated, as there was no detectable LD between them. The frequency distributions of genotypes were evaluated to ensure Hardy-Weinberg equilibrium for all SNPs in the populations [32]. Statistical analyses for ELISA assays were performed using Wilcoxon's U test, on software R version 3.5.2. To avoid the effect of biases such as smoking, smokers and nonsmokers were also analyzed separately. It was considered significant for all tests *P-value* <0.05.

## Results

The characteristics of the studied population are described in Table 1. No significant differences were observed in the groups according to age and gender, suggesting adequate pairing between cases and controls. The covariate smoking habit showed significant difference when the PD and control groups were compared. All results from the comparisons between the groups were adjusted to remove the effects of covariates acting as confounding biases.

**Table 1:** General characteristics of patients with periodontitis (PD) and controls.

Total	Nonsmokers (n = 285)	Smokers (n = 139)	*P* ^*2*^	Female (n = 233)	Male (n = 191)	*P* ^*2*^	Age ^1^	*P* ^*2*^
PD (n = 215)	129 (45%)	86 (62%)	**0.001**	111 (48%)	104 (52%)	0.16	47.6 ±9.4	0.44
Controls (n = 209)	156 (55%)	53 (38%)		122 (58%)	87 (42%)		45.7 ±8.9	

^1^ Age in years, given by mean (±Standard deviation). Student’s t-test.

^2^ Chi-square test.

### Distribution of allele and genotype frequencies

The distribution of genotype frequencies for all studied polymorphisms in the control groups is in accordance with the expected for Hardy-Weinberg equilibrium. The distribution of alleles and genotype frequencies for the three studied polymorphisms is shown in Table 2. No differences were observed in the allele and genotype frequencies between PD and controls for all polymorphisms evaluated. For these SNPs, no statistically significant association was observed between allele variants and PD under analysis in Haploview.

**Table 2:** Distribution of genotype and allele frequencies for *IL16* polymorphisms in the periodontitis (PD) patients compared to controls (smokers plus nonsmokers’ individuals).

Alleles ^1^	PD (n = 215)	Controls (n = 209)	Genotype ^1^	PD (n = 215)	Controls (n = 209)
*rs11556218 T>G*					
*T*	333 (77%)	310 (74%)	*T/T*	122 (57%)	112 (54%)
*G*	97 (23%)	108 (26%)	*T/G*	89 (41%)	86 (41%)
			*G/G*	4 (2%)	11 (5%)
*rs4778889 T>C*					
*T*	341 (79%)	333 (80%)	*T/T*	136 (63%)	135 (65%)
*C*	89 (21%)	85 (20%)	*T/C*	69 (32%)	63 (30%)
			*C/C*	10 (5%)	11 (5%)
*rs4072111 C>T*					
*C*	378 (88%)	373 (89%)	*C/C*	164 (76%)	168 (80%)
*T*	52 (12%)	45 (11%)	*C/T*	50 (23%)	37 (18%)
			*T/T*	1 (1%)	4 (2%)

^1^ No significant difference was observed in allele and genotype frequency distribution between periodontitis and controls in the chi-square test (*P*>0.05).

### Stratified analyses of the results

The data were analyzed in a stratified form, with OR adjustment, in order to remove the possible effects of covariates in the evaluation of associations. Therefore, the values were adjusted for age and gender. The groups of smokers and nonsmokers were analyzed separately and only the significant results are presented in Table 3 (no significant results are presented in S2 Table).

**Table 3:** SNP *rs11556218* genotype frequency distributions between periodontitis (PD) and controls, considering interaction with covariates sex and age.

Model		Genotype	PD (%)	Controls (%)	OR (95% CI) ^1^	*P*	*Pc* ^*2*^	AIC ^4^
Nonsmokers (n = 285)			(n = 129)	(n = 156)				
	Codominant	*T/T*	77 (59.7%)	75 (48.1%)	Ref. ^3^	**0.006**	**0.019**	**387.4**
		*T/G*	51 (39.5%)	71 (45.5%)	0.68 (0.42–1.11)			
		*G/G*	1 (0.8%)	10 (6.4%)	**0.10 (0.01–0.71)**			
	Dominant	*T/T*	77 (59.7%)	75 (48.1%)	Ref. ^3^	**0.039**	0.117	391.3
		*T/G-G/G*	52 (40.3%)	81 (51.9%)	**0.60 (0.37–0.98)**			
	Recessive	*T/T-T/G*	128 (99.2%)	146 (93.6%)	Ref. ^3^	**0.005**	**0.016**	387.7
		*G/G*	1 (0.8%)	10 (6.4%)	**0.10 (0.01–0.83)**			
	Overdominant	*T/T-G/G*	78 (60.5%)	85 (54.5%)	Ref. ^3^	0.290	0.870	394.4
		*T/G*	51 (39.5%)	71 (45.5%)	0.77 (0.48–1.25)			
	Log-additive	---	---	---	**0.56 (0.37–0.86)**	**0.007**	**0.022**	388.3
Smokers (n = 138)			(n = 85)	(n = 53)				
	Codominant	*T/T*	44 (51.8%)	37 (69.8%)	Ref. ^3^	0.085	0.255	187.4
		*T/G*	38 (44.7%)	15 (28.3%)	**2.17 (1.03–4.57)**			
		*G/G*	3 (3.5%)	1 (1.9%)	3.36 (0.32–35.58)			
	Dominant	*T/T*	44 (51.8%)	37 (69.8%)	Ref. ^3^	**0.029**	0.087	185.6
		*T/G-G/G*	41 (48.2%)	16 (30.2%)	**2.23 (1.07–4.64)**			
	Recessive	*T/T-T/G*	82 (96.5%)	52 (98.1%)	Ref. ^3^	0.420	1.260	189.7
		*G/G*	3 (3.5%)	1 (1.9%)	2.46 (0.24–25.55)			
	Overdominant	*T/T-G/G*	47 (55.3%)	38 (71.7%)	Ref. ^3^	0.052	0.156	186.6
		*T/G*	38 (44.7%)	15 (28.3%)	2.05 (0.98–4.29)			
	Log-additive	---	---	---	**2.08 (1.06–4.07)**	**0.028**	0.084	185.5

^1^ OR: *Odds Ratio*; CI = Confidence interval 95%.

^2^ Pc: Bonferroni Correction of *P-value*.

^3^ Ref: Reference (OR = 1.00).

^4^ AIC: Akaike Information Criteria.

In nonsmokers, the variant of the *IL16 rs11556218* was statistically associated with a protection for PD in codominant, dominant, recessive and log-additive inheritance models (Table 3). The codominant inheritance model was selected because it presented the lowest AIC. Individuals with the *G/G* genotype were less susceptible to developing PD compared to individuals with the *T/T* genotype (OR = 0.10; *P* = 0.006). The statistical significance for this model was maintained after BC (*Pc* = 0.019). In smokers, this same polymorphism (*rs11556218*) was statistically associated with the risk for PD in the dominant and log-additive inheritance models (Table 3). The log-additive was considered the best model (OR = 2.08; *P* = 0.028) according to the lowest AIC value; however, significance was lost after BC (*Pc* = 0.084).

SNPs *rs4778889* and *rs4072111* were not significantly associated with PD in this study when smokers and nonsmokers were analyzed separately. Differences were also not observed between case and controls when the age covariate was considered for the analysis of all polymorphisms evaluated.

Six possible haplotypes were obtained for the SNPs *rs11556218(T>G)_rs4072111(C>T)_rs4778889(T>C)* and the haplotype frequencies observed for the control group were: *TCT* 59.2%, *GCT* 11.7%, *GCC* 11.7%, *TTT* 6.1%, *TCC* 7.0% and *GTT* 3.0%. In PD patients, the haplotype frequency of *TTT* (11.3%) remained significant after testing with 10,000 permutations with haplotypes and single markers (*Pc* = 0.03). In PD patients, the haplotype frequency of *TTT* (11.3%) was higher than in controls (OR = 2.45; 95% CI = 1.18–5.08; P = 0.01) and remained significant after testing with 10,000 permutations with haplotypes and single markers (*Pc* = 0.03). The distribution of the other haplotype frequencies was similar between PD patients and controls.

### Serum IL-16 measurement

**Fig 1:**
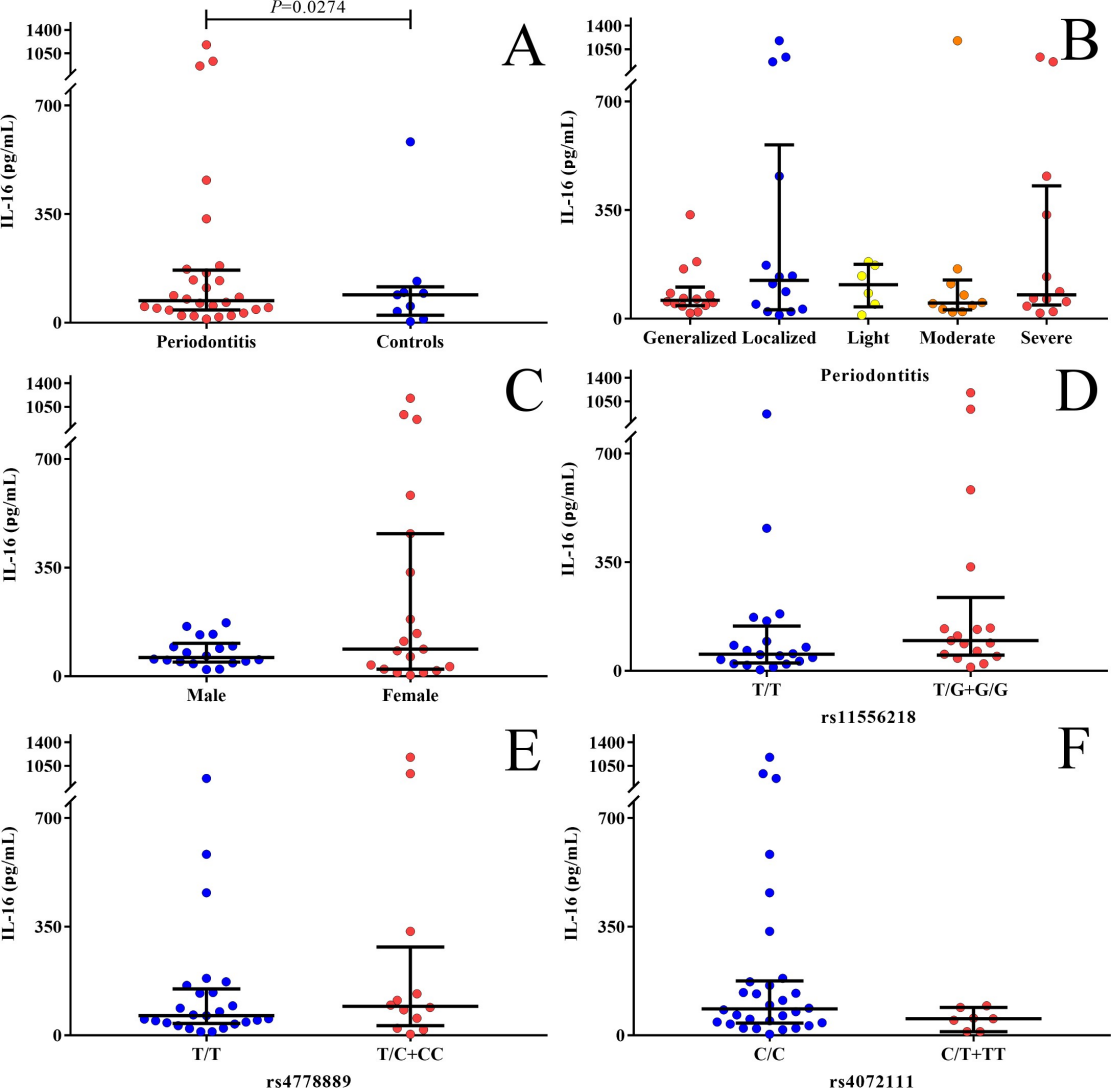
Serum levels of IL-16 in nonsmoking PD patients and controls. (A) Comparison of IL-16 serum level between PD and controls; (B) between periodontitis groups; (C) between men and women with periodontitis; (D) among patients with and without *G* allele variant of *rs11556218* for *IL16*; (E) among patients with and without the *C* allele variant of *rs4778889* for *IL16*; and (F) among patients with and without the variant allele *T* of *rs4072111* for *IL16*.

Thirty-nine nonsmokers were selected to determine IL-16 serum levels (9 controls and 30 PD patients, 20 men and 19 women). PD patients were classified according to the extent of the disease (localized n = 15 and generalized n = 15) and the degree of severity (mild n = 6, moderate n = 10, and severe n = 14). Lower serum levels for IL-16 were found in individuals with PD (median: 70.7 pg/ml) when compared to controls (89.4 pg/ml, *P* = 0.027) (Fig 1A). No significant differences were observed in serum levels for IL-16 between the forms of the disease (Fig 1B) or between females and males (Fig 1C). There were no statistically significant differences in the IL-16 serum levels when compared to the genotypes of *rs11556218 T>G* (Fig 1D), *rs4778889 T>C* (Fig 1E) and *rs4072111 C>T* (Fig 1F).

## Discussion

The main finding of this study was that the *rs1556218* polymorphism, located in exon 6 of *IL16*, was associated with a lower risk for developing PD, regardless of other risk factors such as smoking and gender. As far as we know, this is the first study that correlates the *rs11556218* variant of *IL16* with PD.

The *G/G* genotype of *IL16 rs11556218 T>G* was associated with PD protection in nonsmokers. This SNP is located in the exon region of *IL16* and the variant allele encodes a protein with modifications in the PDZ2 domain, which is responsible for structural changes in *IL-16* that affect its function in the immune response [34]. Thus, we hypothesize that this genetic association cannot be correlated with serological levels of *IL-16*, because the polymorphism of *rs11556218* does not directly affect the amount of cytokine produced. In fact, we did not found difference between *IL-16* genotypes and IL-16 serum levels in PD studied population. Polymorphisms on genes from other cytokine, such as IL-1α, IL-1β, TNFα and IL-10, has influence in PD [35], suggesting that the presence of pro-inflammatory cytokines (and inhibition of anti-inflammatory cytokines) direct the polarization of the immune response profiles (Th1 and Th17 were predominant in PD) and, consequently, in the disorders of tissue repair in the periodontium [36]. Although it is not yet clear what effect the IL16 polymorphism has on the development of PD, it is possible that it’s related to less tissue aggression and maintenance of bone homeostasis: the structural modification of IL-16 would compromise its function in chemotaxis on CD4+ cells and the production of inflammatory cytokines by these cells which would lead to a modulation of the immune response in the oral microenvironment. Regarding diseases with bone compromise, IL-16-mediated protection was reported [18, 20].

When smokers were separately analyzed, the *IL16 rs11556218* variant was associated with the risk of PD, although this correlation was not confirmed after Bonferroni correction. In addition to the genetic component, environmental factors confer an important risk for the development of PD and, among these, tobacco use is recognized as one of the main risk factors for this disease [37]. Unfortunately, in our study, it was not possible to test the association of IL-16 levels with PD in smokers. However, according to Laan et al. [38], IL-16 airway levels are increased in smokers, and this may influence systemic immune modulation by altering the number and responsiveness of CD4+ cells. In general, the alteration caused by tobacco on the gene expression of mononuclear cells from the peripheral blood seems to have greater destructive inflammatory effects [39]. Therefore, the combination of smoking and genetic polymorphisms may alter the regulation of the immune response in PD. This fact may be responsible for the non-association between the polymorphism of the *IL16 rs11556218* and PD when all patients (smokers and nonsmokers) were evaluated.

*IL16 rs11556218* polymorphism has been associated with other diseases. With regard to diseases with alteration of bone homeostasis, the protective effects of this polymorphism were observed in knee osteoarthritis [18, 20], which may be the result of changes in IL-16 activity altering the effects of chronic inflammation on the synovial membrane. This variant was also associated with the risk of osteoporosis in postmenopausal women, although there was no significant alteration in serum cytokine levels [40]. This SNP has also been associated with many types of cancer [41]. In patients with osteosarcoma, *IL16* variants were related to higher levels of IL-16 and, consequently, in the effects in the production of other tumor-related inflammatory cytokines, such as TNF-α and IL-1β [42].

The polymorphism *rs4778889* of IL16, also known as *T-295C* and located in the promoter region of the gene, was not significantly associated with PD in this study. Similar results were found in two previous studies conducted in 2005 and 2018 in an Iranian adult population with PD [23, 24]. An association was also not found between this SNP and other diseases, such as asthma [43, 44], osteosarcoma [42], multiple sclerosis [45], endometriosis [46] and autoimmune thyroid disease [47]. On the other hand, this polymorphism was associated with an increased risk for Graves' disease [48] and systemic lupus erythematosus [49].

The *IL16 rs4072111* SNP was not associated with PD in this study in any of the subgroups evaluated. Nevertheless, the *TTT* haplotype (*rs11556218_rs4072111_rs4778889*) of *IL16*, containing an allele variant only in *rs4072111 (C>T)*, was associated with a higher risk of developing PD, although no significant linkage disequilibrium (LD) has been found among these SNPs. Previous studies have not observed an association between *rs4072111* and osteoporosis [40], osteosarcoma [42], endometriosis [34] and Graves' disease [50]. However, this polymorphism was a risk factor for systemic erythematic lupus among Chinese [49] and multiple sclerosis in Iranians [45].

In relation to IL-16 serum levels, lower values were observed in PD patients when compared to controls. But, serum levels for IL-16 were similar when patients were classified in different clinical forms of the disease, though there was a tendency for IL-16 serum levels to decrease in the most generalized and severe forms of PD. These results were similar to those reported in a study on IL-16 levels in crevicular fluid (GCF) of PD patients [14] where a low level of this cytokine was found. The authors suggested that IL-16 may play a protective role in the periodontium or be suppressed in PD [14]; however, these relationships have not yet been clarified.

This study has its limitations. The main limitation of our study concerns the statistical power of some comparisons. There is a low statistical power of analysis when some subgroups were analyzed, which can limit the ability to detect associations in these specific subgroups. In addition, we were unable to measure IL-16 in smokers and in tissues related to the local immune response involved in PD, such as GCF. The positive points of this study were the good clinical selection of the participants and the rigorous inclusion and exclusion criteria adopted for cases and controls. In addition, multivariate and stratified analyses were performed for important factors of predisposition for PD that could be confusing in analyzes, such as smoking, gender and age. Correction for multiple tests (BC and permutation test) were applied. Also, in addition to the polymorphism of the promoter region of *IL16 (rs4778889)* previously studied for PD [23, 24] we evaluated the effect of two other SNPs with missenses modifications *(rs11556218* and *rs4072111)* in this disease.

## Conclusions

The *IL16 rs11556218* polymorphism and the IL-16 serum level were associated with protection for periodontitis in a nonsmoking Brazilians sample, although they seem to be independent events. No association was observed between polymorphisms in *IL16 rs4778889* and *rs4072111* and periodontitis. Environmental factors that alter the immunopathogenesis of periodontitis, such as smoking, influenced the association of *IL16* with PD.

## Supporting information

S1 Table Self-declared ethnic characterization of patients with periodontitis (PD) and controls. (DOCX)

S2 Table *IL16 rs4778889* and *rs4072111* genotype frequency distributions between periodontitis (PD) and controls, considering interaction with covariates sex and age. (DOCX)
