# Supplementary material for: Association of IL16 polymorphisms with periodontitis in Brazilians: A case- control study
Source: PLoS One. 2020 Sep 11;15(9):e0239101. doi: 10.1371/journal.pone.0239101 (PMC7485854; doi:10.1371/journal.pone.0239101)
Supplement: S1 Table — (DOCX) [file pone.0239101.s001.docx]

**Supplementary Table 1. Self-declared ethnic characterization of patients with periodontitis (PD) and controls.**

| **Ethnic group** | **Total (%)** | **PD (%)** | **Control (%)** | ***P*** |
| --- | --- | --- | --- | --- |
|  | **(n=424)** | **(n=215)** | **(n=209)** |  |
| White | 267 (63.0) | 140 (65.1) | 127 (60.8) | 0.38^1^ |
| Mulatto | 97 (22.9) | 52 (24.2) | 45 (21.5) | 0.53^1^ |
| Black | 47 (11.0) | 18 (8.4) | 29 (13.9) | 0.07^1^ |
| Amerindians | 2 (0.4) | 0 (0.0) | 2 (1.0) | 0.97^2^ |
| No response | 11 (2.6) | 5 (2.3) | 6 (2.9) |  |

^1^ Chi-square test.

^2^ Fisher's exact test.
