# Supplementary material for: Association of IL16 polymorphisms with periodontitis in Brazilians: A case- control study
Source: PLoS One. 2020 Sep 11;15(9):e0239101. doi: 10.1371/journal.pone.0239101 (PMC7485854; doi:10.1371/journal.pone.0239101)
Supplement: S2 Table — (DOCX) [file pone.0239101.s002.docx]

**Supplementary Table 2. *IL16* *rs4778889* and *rs4072111* genotype frequency distributions between periodontitis (PD) and controls, considering interaction with covariates sex and age.**

| **SNP/Model** | | **Genotype** | **PD (%)** | **Controls (%)** | **OR (95% CI) ^1^** | ***P*** | ***Pc ^2^*** | **AIC ^4^** |
| --- | --- | --- | --- | --- | --- | --- | --- | --- |
| Nonsmokers (n = 285) | ***rs4778889 T>C*** | | (n = 129) | (n = 156) |  |  |  |  |
|  | Codominant | *T/T* | 77 (59.7%) | 97 (62.2%) | Ref. ^3^ | 0.900 | 1.000 | 397.3 |
|  |  | *T/C* | 43 (33.3%) | 48 (30.8%) | 1.13 (0.67-1.88) |  |  |  |
|  |  | *C/C* | 9 (7.0%) | 11 (7.0%) | 1.00 (0.39-2.56) |  |  |  |
|  | Dominant | *T/T* | 77 (59.7%) | 97 (62.2%) | Ref. ^3^ | 0.690 | 1.000 | 395.4 |
|  |  | *T/C-C/C* | 52 (40.3%) | 59 (37.8%) | 1.10 (0.68-1.79) |  |  |  |
|  | Recessive | *T/T-T/C* | 120 (93.0%) | 145 (93.0%) | Ref. ^3^ | 0.930 | 1.000 | 395.5 |
|  |  | *C/C* | 9 (7.0%) | 11 (7.0%) | 0.96 (0.38-2.42) |  |  |  |
|  | Overdominant | *T/T-C/C* | 86 (66.7%) | 108 (69.2%) | Ref. ^3^ | 0.640 | 1.000 | 395.3 |
|  |  | *T/C* | 43 (33.3%) | 48 (30.8%) | 1.13 (0.68-1.87) |  |  |  |
|  | Log-additive | --- | --- | --- | 1.06 (0.72-1.54) | 0.780 | 1.000 | 395.5 |
| Smokers (n = 138) | ***rs4778889 T>C*** | | (n = 85) | (n = 53) |  |  |  |  |
|  | Codominant | *T/T* | 58 (68.2%) | 38 (71.7%) | Ref. ^3^ | 0.620 | 1.000 | 191.4 |
|  |  | *T/C* | 26 (30.6%) | 15 (28.3%) | 1.09 (0.51-2.34) |  |  |  |
|  |  | *C/C* | 1 (1.2%) | 0 (0%) | - |  |  |  |
|  | Dominant | *T/T* | 58 (68.2%) | 38 (71.7%) | Ref. ^3^ | 0.750 | 1.000 | 190.3 |
|  |  | *T/C-C/C* | 27 (31.8%) | 15 (28.3%) | 1.13 (0.53-2.42) |  |  |  |
|  | Recessive | *T/T-T/C* | 84 (98.8%) | 53 (100%) | Ref. ^3^ | 0.340 | 1.000 | 189.5 |
|  |  | *C/C* | 1 (1.2%) | 0 (0%) | - |  |  |  |
|  | Overdominant | *T/T-C/C* | 59 (69.4%) | 38 (71.7%) | Ref. ^3^ | 0.860 | 1.000 | 190.3 |
|  |  | *T/C* | 26 (30.6%) | 15 (28.3%) | 1.07 (0.50-2.30) |  |  |  |
|  | Log-additive | --- | --- | --- | 1.18 (0.57-2.46) | 0.660 | 1.000 | 190.2 |
| Nonsmokers (n = 285) | ***rs4072111 C>T*** | | (n = 129) | (n = 156) |  |  |  |  |
|  | Codominant | *C/C* | 102 (79.1%) | 124 (79.5%) | Ref. ^3^ | 0.360 | 1.000 | 395.5 |
|  |  | *C/T* | 26 (20.1%) | 28 (17.9%) | 1.09 (0.60-1.99) |  |  |  |
|  |  | *T/T* | 1 (0.8%) | 4 (2.6%) | 1.00 (0.39-2.56) |  |  |  |
|  | Dominant | *C/C* | 102 (79.1%) | 124 (79.5%) | Ref. ^3^ | 0.930 | 1.000 | 395.5 |
|  |  | *C/T-T/T* | 27 (20.9%) | 32 (20.5%) | 0.98 (0.54-1.75) |  |  |  |
|  | Recessive | *C/C-C/T* | 128 (99.2%) | 152 (97.4%) | Ref. ^3^ | 0.160 | 0.480 | 393.6 |
|  |  | *T/T* | 1 (0.8%) | 4 (2.6%) | 0.24 (0.03-2.22) |  |  |  |
|  | Overdominant | *C/C-T/T* | 103 (79.8%) | 128 (82.0%) | Ref. ^3^ | 0.710 | 1.000 | 395.4 |
|  |  | *C/T* | 26 (20.2%) | 28 (18.0%) | 1.12 (0.61-2.04) |  |  |  |
|  | Log-additive | --- | --- | --- | 0.88 (0.53-1.49) | 0.640 | 1.000 | 395.3 |
| Smokers (n = 138) | ***rs4072111 C>T*** | | (n = 85) | (n = 53) |  |  |  |  |
|  | --- | *C/C* | 62 (72.9%) | 44 (83.0%) | Ref. ^3^ | 0.190 | 0.570 | 188.6 |
|  |  | *C/T* | 23 (27.1%) | 9 (17.0%) | 1.76 (0.74-4.20) |  |  |  |

^1^ OR: *Odds Ratio*; CI = Confidence interval 95%.

^2^ Pc: Bonferroni Correction of *P-value*.

^3^ Ref: Reference (OR = 1.00).

^4^ AIC: Akaike Information Criteria.
